# Low Serum Urate Levels Are Associated to Female Gender in Multiple Sclerosis Patients

**DOI:** 10.1371/journal.pone.0040608

**Published:** 2012-07-27

**Authors:** Stefano Zoccolella, Carla Tortorella, Pietro Iaffaldano, Vita Direnzo, Mariangela D’Onghia, Elena Luciannatelli, Damiano Paolicelli, Paolo Livrea, Maria Trojano

**Affiliations:** Department of Neurosciences and Organs of Senses of the University of Bari, Bari, Italy; Innsbruck Medical University, Austria

## Abstract

**Background:**

Urate is a natural antioxidant and may prevent CNS tissue damage and the clinical manifestations of experimental autoimmune encephalitis. Results from clinical studies are conflicting and the contribution of urate to the pathogenesis of Multiple Sclerosis (MS) remains uncertain.

**Objective:**

To evaluate serum urate levels in MS patients and their relationships with clinical, demographic and MRI variables.

**Methods:**

Levels of non-fasting serum uric acid and creatinine were determined by an automated enzymatic assay and glomerular filtration rate was assessed in 245 MS patients, in 252 age/sex-matched neurological controls (NC) and in 59 Healthy controls (HC).

**Results:**

Median serum urate levels did not differ between MS patients (3.8 mg/dL), HC (4.0 mg/dl) and NC (4.0 mg/dL). Serum urate levels were lower in females than in males in all groups (p = <0.0001). In female-MS, serum urate levels (3.2 mg/dL) were lower compared to those in female HC (3.8; p = 0.01) and NC (3.5 mg/dL; p = 0.02), whereas in male-MS they(4.8 mg/dL) did not differ from those in male HC (4.5 mg/dl) and NC (4.8 mg/dL). Urate concentrations trended to be lower in Clinically isolated syndromes suggestive of MS (3.7 mg/dL) and in relapsing MS (3.7 mg/dL), compared to patients with progressive MS (4.4 mg/dL; p = 0.06), and in patients with an annual relapse rate (ARR) >2 (3.3 mg/dL) than in those with an ARR ≤2: 3.9 mg/dL; p = 0.05). Significant lower serum urate levels were found in females than in males in all clinical MS subtypes (p<0.01), separately evaluated. Female sex (beta: −0.53; p<0.00001) was the most significant determinant of serum urate concentrations in MS patients on multivariate regression analysis.

**Conclusions:**

Our findings suggest that low urate levels could be of significance in predominantly inflammatory phases of MS even at the early stage and mainly in females.

## Introduction

Multiple Sclerosis (MS) is a neurologic disease of unknown aetiology characterized by inflammation, demyelination and diffuse axonal degeneration throughout the central nervous system. [1]

Uric acid is the main end-product of purine metabolism and circulates in humans at high concentrations, near the limits of its solubility. [2], [3] In vitro and in vivo studies showed that urate may protect neurons against oxidative damage caused by reactive nitrogen and oxygen species, particularly by peroxynitrite. [4], [5] Peroxynitrite along with other free radicals are believed to be involved in the inflammation, demyelination and axonal injury that occur in MS. [5]–[7] Furthermore, epidemiological studies conducted in US on more than 20 million patient records revealed that MS and gout are almost mutually exclusive. [8] All these observations raised the possibility that hyperuricemia may play a protective role against MS.

In the last ten years several studies evaluated serum urate levels in patients with MS, reporting conflicting results; [8]–[27] some case-control studies found lower, [8]–[15] but others reported higher [16]–[18] serum urate levels in MS patients than in neurological [16], [19], [20] and healthy controls [10], [16]–[19]; finally, other studies found no difference between the MS and neurological, [21], [22] and healthy controls. [23]–[27] A recent metanalysis was in favour of lower serum urate levels in MS than in healthy subjects and neurological controls [28] Furthermore, in a recent clinical trial, the combination of interferon beta and inosine was safe and well tolerated but did not provide any additional benefit on accumulation of disability compared with interferon beta alone. [29] Based on these observations, the effective contribute of urate to the pathogenesis of MS still remains unclear.

In this cross-sectional study we evaluated non-fasting serum levels of uric acid and glomerular filtration rate in MS patients and in age and sex matched healthy (HC) and neurological controls (NC). Potential associations between urate levels and demographic, clinical, laboratory and magnetic resonance imaging (MRI) variables were also assessed in MS group.

## Methods

### Study Population

We enrolled all consecutive patients with a diagnosis of Clinically Isolated Syndromes (CIS) suggestive of MS, or of MS, according to the revised Mc Donald’s criteria of 2005, [30] and 2010 [31], referring for their first visit to the neurology clinics of the Department of Neuroscience and Sense Organs of the University of Bari, Italy, between January 1, 2007 and July 30, 2010. The population consisted of 58 CIS, 148 relapsing remitting (RR), 30 secondary progressive (SP) and 9 primary progressive (PP) MS. None of patients received corticosteroid treatment in the previous two months and only seven percent of them assumed immunomodulatory therapy.

NC (n = 252) and HC (n = 59) were matched by sex and age with the MS patients. HC were healthy volunteers, whereas NC were recruited during the same time period among patients who were admitted to the same neurological clinics for symptoms as vertigo, dizziness, syncope, radicular or non radicular pain, or minor head trauma. After a complete neurological assessment and central and peripheral diagnostic tests, all patients were classified as having labyrinthitis (n = 64), neurosis (n = 92), radiculopathy (n = 27), vasovagal syncope (n = 37) and minor head trauma (n = 32).

All subjects gave their written informed consent to the study, which was approved by the institutional review board of the University of Bari.

We excluded subjects with a medical history of comorbidities (stroke, myocardial infarction, renal or hepatic dysfunctions, dementia, Parkinson disease and other neurodegenerative disorders), and patients with acute medical conditions (infections and surgical interventions) and alcohol abuse, in the previous three months. [31] Finally, subjects who assumed medications that can either increase (as ascorbic acid, cisplatin, diazoxide, diuretics, epinephrine, ethambutol, levodopa, methyldopa, phenothiazines, and theophylline) or decrease (as high-dose aspirin, azathioprine, clofibrate, corticosteroids, estrogens, glucose infusion, guaifenesin, mannitol, probenecid, and warfarin) urate concentrations were excluded from the study. [32] Based on these criteria, we excluded 10 patients: 3 for vascular disease, 1 for renal dysfunction, 2 for recent surgical interventions, 4 because were receiving medications that can interfere with urate acid levels.

MS patients, NC and HC were caucasic in origin and resident in the same geographic area. No subject was vegetarian.

Levels of non-fasting serum uric acid and creatinine were determined using an automated enzymatic assay. Kidney function was evaluated using an estimate of the glomerular filtration rate, based on the formula proposed by Levey et al, which incorporates serum creatinine, age, sex and race. [33]

Disability status was assessed using the Expanded Disability Status Scale (EDSS) score. [34] Annualised relapse rate (ARR) from disease onset was calculated for each patient. Paraclinical disease activity was investigated in the patients by brain and spinal cord gadolinium (Gd)-MRI scans within 30 days from blood sample collection. Gd-MRI coeval to serum samples was available in 228 of 245 MS patients (93%). MS patients were considered to have MRI disease activity when they had one or more Gd-enhancing lesions.

### Statistical Analysis

A natural log transformation of the parametric data was carried out to better meet the assumption of normality, because urate levels were not normally distributed. Student’s t-test and analysis of variance (ANOVA) were used for parametric data, and chi-square and Fisher’s test were used to analyze non-parametric data. Post-Hoc analysis with Bonferroni’s test were performed for multiple comparison of Urate levels in MS (either overall and classified according to clinical phenotype), HC and NC stratified by gender.

We studied the association between MS/NC status and urate levels in several logistic regression models, using serum urate levels as both a continuous and a categorical covariate in the model. When urate levels were considered as a categorical variable, we stratified their distribution into tertiles, using the bottom tertile as the reference group. Because of the different distribution of urate levels across gender we also calculated sex-specific tertiles. In the multivariate analyses, data were adjusted for age, sex, and glomerular filtration rate.

Simple and multivariate linear regressions were used to study the association between urate levels and glomerular filtration rate, age, sex, disease phenotype (CIS, RR, SP,PP), disease duration, ARR, EDSS score and MRI disease activity, in MS patients. In all statistical analyses, the level of statistical significance was set at p = 0.05. All statistical analyses were performed with SPSS version 16.0 for Windows (SPSS Inc., Chicago, IL, USA).

## Results

The study was carried out on 245 MS patients (94 males, 151 females), 252 NC (99 males and 153 females) and 59 HC (21 males, 38 females). In MS group, median disease duration was 25.3 months (range: 0.4–406.1), median EDSS score was 2.5 (1–8). Median ARR in patients with RR and SP MS was 0.7 (0.06–5.7). Seventy-eight patients (34%) showed at least one MRI Gd enhancing lesion. Thirty-one percent (77) was in a relapsing phase.

No significant differences were found in median age (MS: 37 years; range: 15–74; NC: 37 years; range: 14–76; HC: 35 years; range: 15–74; p = 0.6) and sex (p = 0.4) distribution and in median glomerular filtration rate (108 versus 103 and 102 ml/min/1.73 m^2^) between the three groups. Mean and median serum urate levels did not significantly differ between MS patients (3.9±1.3 and 3.8 mg/dL), NC (4.1±1.3 and 4.0 respectively) and HC (4.0±1.2 and 4.0 mg/dl). Multivariate logistic analyses showed that, even if higher urate levels were less likely associated with MS (age, sex and kidney function adjusted odds ratio [OR] for the MS diagnosis: 0.7; 95% CI: 0.4–1.4), this finding was not statistically significant (p = 0.26); similar results were obtained considering urate levels as a categorical variable (age, sex, and kidney function adjusted OR for MS diagnosis for the highest [>4.4 mg/dl] versus the lowest tertile of urate levels [≤3.3]): 0.7 :95% CI: 0.4–1.2; p for trend: 0.18).

**Figure 1:**
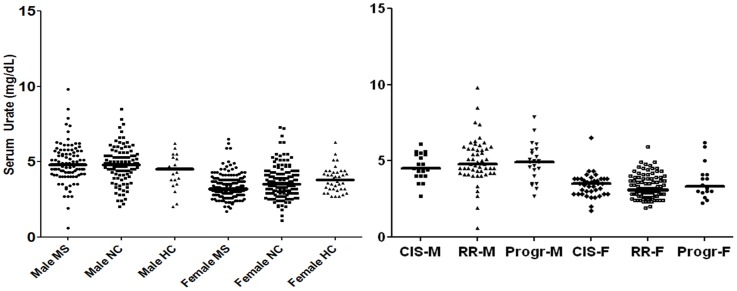
Serum Urate levels in our study cohort (MS patients, NC and HC), stratified by sex (A) and clinical phenotype(B). Figure legend. Median serum urate levels were lower in female MS (3.2 mg/dL) compared to female NC (3.5 mg/dL), and female HC (3.8 mg/dL; p = 0.004; Bonferroni’s test: female MS versus female NC p = 0.02; female MS versus female HC p = 0.01). Moreover, serum urate was lower in female with RR MS (3.1 mg/dL; compared to female NC (Bonferroni’s test: p = 0.03) and female HC (Bonferroni’s test: p = 0.04).

After stratification of MS, NC and HC groups by gender (Figure 1 A), serum urate levels were lower in females than in males in all groups (p<0.0001). However in female MS, serum urate levels (3.2 mg/dL) were lower compared to those in female HC (3.8 mg/dL; Bonferroni’s test: 0.01) and NC (3.5 mg/dL; Bonferroni’s test: 0.02), whereas in male MS (4.8 mg/dL) they did not differ from those in male HC (4.5 mg/dl) and NC (4.8 mg/dL). (Figure 1 A) Glomerular filtration rate was similar across genders either in MS or in HC and NC groups. To rule out the possible confounding effects of the outliers, we also performed a sensitivity analysis excluding subjects with the lowest urate concentrations (<2 mg/dL). The analysis confirmed lower median urate levels in female MS patients (n = 149; 3.2 mg/dL, range: 2–6.5; p = 0.003), compared to female HC (n = 38; 3.8 mg/dL; range: 2.7–6.3; Bonferroni’s test: 0.02) and NC (n = 150; 3.6 mg/dL; range 2–7.3; Bonferroni’s test: p = 0.01).

Logistic regression models revealed that low urate levels trended to be associated with female MS. The age, sex, and kidney function adjusted OR for female MS for the highest [>3.8 mg/dL] versus the lowest tertile [≤3 mg/dL] of serum urate levels was 0.4 (95% CI: 0.2–0.7; p for trend: 0.002).

We also evaluated serum urate levels in MS patients, stratifying MS according to clinical phenotypes. Urate concentrations in CIS patients (3.7 mg/dL, range: 1.7–6.5) and in RR MS (3.7 mg/dL, range: 0-6-9.8) were lower compared to patients with progressive MS (4.4 mg/dL, range: 2.2–7.9; p = 0.05). However, the difference was not significant when compared to HC (4.0 mg/dL) and NC (4.0 mg/dl). Stratifying MS subtypes by gender, (Figure 1 B) the difference in serum urate concentrations between males and females was found in all clinical subtypes (CIS females: 3.5 mg/dL, range: 1.7–6.5, CIS males: 4.5 mg/dL, range: 2.7–6.1; p<0.0001; RR females: 3.1 mg/dL range: 1.9–5.9, RR males:4.8 mg/dL, range: 0.6–9.8; p<0.0001; progressive MS females: 3.3 mg/dL, range: 2.2–6.2, progressive MS males: 4.9 range: 2.7–7.9; p = 0.003). Finally, median urate levels resulted significantly lower in female with RR MS compared with female HC (Bonferroni’ s test: p = 0.04) and female NC (Bonferroni’ s test: p = 0.03).

In Table 1 the distribution of clinical and MRI characteristics in MS patients across gender is shown. Male MS, as expected, presented, more frequently, a progressive course (chi square: 8.4; p = 0.01) and an EDSS score >4 (chi square: 8; p = 0.002) than female MS, whereas a higher percent of female MS showed an ARR >2 (chi square: 3; p = 0.04) than male MS. When we looked at the relationships between clinical and MRI variables and urate levels in MS we found that serum urate was lower in patients with an ARR >2 (3.3 mg/dL; range: 1.9–9.8), compared to patients with an ARR ≤2 (3.9 mg/dL, range: 0.6–8.5; p = 0.05). Serum urate did not differ between in patients with disease duration (>25.3 months: 3.9 mg/dL versus ≤25.3 months: 3.7 mg/dL; p = 0.1) with EDSS score (≤4: 3.8 mg/dL versus >4: 3.8 mg/dL) with the presence (3.8 mg/dL) or absence (3.8 mg/dL) of Gd+ MRI lesions and with the immunomodulatory treatment (3.6 mg/dL versus untreated patients: 3.8 mg/dL; p>0.5;).

Multivariate regression model (Table 2) revealed that female sex (beta: −0.53; p<0.00001) was the most significant determinant of serum urate concentrations in MS patients, after adjustment for age(beta: −0.27; p = 0.003), glomerular filtration rate(r: −0.003; p = 0.0002), clinical phenotype, disease duration, ARR, disease activity and disability level.

## Discussion

The most relevant finding of this cross-sectional study is that low serum urate levels are associated with female MS predominantly at the early stage and during the relapsing phase of the disease. In fact we found that serum urate levels were significantly lower in female MS compared to males MS and also to female HC and NC. In addition we demonstrated lower serum urate levels in clinical phenotypes with more prominent inflammation, such as CIS and RR MS, and the lowest levels in RR female MS, which were more frequently associated with higher ARR in comparison with RR male MS. Moreover multivariate analyses clearly showed that female sex was the most significant determinant of serum urate concentrations in MS, after adjustment for demographic, clinical, MRI variables and glomerular filtration rate.

**Table 1:** Distribution of clinical and MRI characteristics in MS patients across gender.

	Males	Females
**Clinical subtype**		
CIS MS	19 (20%)	39 (26%)
RR MS	52 (55%	96 (64%)
Progressive MS	23 (25%)	16 (10%)°
Frequency of ARR >2	12/57 (17%)	33/110(30%)°°
Frequency of EDSSscore >4	26 (28%)	19 (13%)°°°

°chi square: 8.4; p = 0.01.

°°chi square: 3; p = 0.04.

°°°chi square: 8; p = 0.002.

These findings are in agreement with previous studies showing that the low serum urate concentrations in MS are associated with female gender [9], [11], [19], [20] and that more pronounced inflammatory findings, such as more frequent relapses and occurrence of Gd-enhancing MRI lesions are more common in female compared to male MS. [28], [35] Although the relatively light difference in median serum levels between female MS and female HC and NC may raise doubt on the clinical significance of our findings they seem to suggest a role of low urate level in the higher susceptibility to develop active RR forms of MS in female sex.

**Table 2:** Multivariate linear regression analysis.

Variable	beta	p
Age	−0.11	0.1
Female sex	−0.53	<0.00001
Glomerular filtration rate	−0.27	0.003
Disease duration	−0.03	0.9
Disease course		
RR versus CIS	0.001	0.9
Progressive versus CIS	0.14	0.2
Disability level (EDSS score ≥4		
versus EDSS <4)	−0.03	0.7
Disease activity	−0.05	0.5
Annual relapse rate >2	−0.06	0.5

A great number of studies evidenced that the neuroinflammation that occurs in several neurologic disorders, including MS, directly induces the production of nitric oxide and superoxide, leading to a vast increase in peroxynitrite formation. [5] Peroxynitrite may play a role in the pathogenesis of MS, both in the demyelination, because of its ability to induce lipid peroxidation of the highly fatty myelin sheaths, [7] and in the axonal damage, by inducing oxidative stress and DNA damage. [5]

Uric acid is a natural anti-oxidant and a peroxynitrite scavenger and accounts for up to 60% of the free radical scavenging activity in human blood. [3] In vitro studies demonstrated that urate levels may reduce the damage to neurons elicited by reactive oxygen species and peroxynitrite and by glutamate excitotoxicity. [3], [5]

Several reports previously investigated serum urate levels in patients with MS compared with neurological and healthy controls, with conflicting results. [8]–[27] Furthermore, most of these studies [12], [16], [18]–[22], [25], [27] were conducted on small cohorts of MS patients and did not control urate levels for significant determinants of serum urate, particularly for renal function. Only one study [15] evaluated serum urate concentrations at the earliest stage of MS (i.e. CIS). A recent metanalysis. [28] demonstrated that serum uratelevels are lower in patients with MS,than in HC, as we found in this paper. However, so far no case-control study assessed serum urate concentrations according to gender.

The correlation between urate levels and clinical and paraclinical disease activity and disability has been also evaluated, with conflicting results [9], [11], [13], [15], [19], [21], [25], [26], [36] Consistently with the results of the metanalysis, [28] in our study serum urate did not correlate with disability level and disease duration, but lower urate levels were observed in female patients with higher disease activity (ARR) and in the early phases (CIS) of the MS. Based on these findings the contribute of inadequate protection against reactive oxygen and peroxynitrite by urate to the axonal loss and the clinical progression of the disease still remains uncertain, but a role of low urate in favouring neuroinflammation and demyelination that occur early in MS and mainly in females may be hypothesized.

Several limitations of our study however deserve discussion. First, the study has a cross-sectional design that prevents us to assess a temporal relationship between the decrease in serum urate levels and MS course; the change in urate concentrations might not be a primary event, but rather represents an epiphenomenon related to its scavenging activity; however the trend for lower urate levels observed in patients with CIS seems to indicate that the decrease in urate concentrations is present since the earliest stage of the disease. Second, this is a clinical based study from a tertiary center and therefore our case-series may not be completely representative of the whole MS population, since cases with a more severe phenotype less likely come to the attention of a tertiary center. Third, we did not use sophisticated analytical methods able to assess other purine compounds such as hypoxanthine, xanthine that could add further useful information about cell energy imbalance, mitochondrial dysfunction and ATP consumption.

Finally, we did not collect information on study participants’ body mass index. Urate levels positively correlate with age, sex, renal function and body mass index, and malnutrition is a major determinant of lower serum urate concentrations. [37] However, a causal correlation between the decrease in uric acid levels observed in our study and malnutrition is unlikely, as we observed the decrease in urate levels in patients with CIS and early RR, whereas malnutrition due to impaired swallowing may occur in MS only in the latest stage of the disease.

In conclusion, our findings favours the view that low urate levels could be of significance in predominantly inflammatory phases of MS even at the early stage and mainly in females.Future longitudinal studies with a prospective design and using more sophisticated analytical methods should be conducted on large cohorts to clarify the effective role of urate in MS, and to evaluate if the change in urate levels may be used to predict the development of the progressive phase of MS.
